# Children’s Adverse Childhood Experiences and Parent-Reported Use of Supplemental Nutrition Assistance Program in U.S. Households

**DOI:** 10.1007/s11414-025-09988-6

**Published:** 2026-01-22

**Authors:** Edson Chipalo, Rebecka Bloomer

**Affiliations:** School of Social Work, College of Allied Health Sciences, University of Cincinnati, Cincinnati, OH USA

## Abstract

Adverse childhood experiences (ACEs) have been linked to food insecurity, poor health outcomes, and socioeconomic challenges. Despite this, there is a dearth of studies connecting ACEs to use of safety net programs in the United States (U.S). This study examines the prevalence and association between ACEs and parent-reported use of SNAP benefits in U.S. households. Data were obtained from the parent-reported 2022 National Survey of Children’s Health ( N = 52,521 children). Descriptive statistics were used to estimate prevalence rates, and six logistic regression models were used to determine the significant association between ACEs and parent-reported SNAP benefits use during the past 12 months. An estimated 57.5% of children with one or more ACEs had parent-reported SNAP benefits use in their households during the past 12 months. Children’s individual ACE exposure such as  experiencing economic hardships (AOR = 2.29) and being discriminated due to health conditions (AOR = 1.69) were associated with higher likelihood of parent-reported SNAP benefits use in households during the past 12 months. However, living with a family member with mental illness was associated with lower likelihood of parent-reported SNAP benefits use in households during the past 12 months (AOR = 0.70). Additionally, children’s exposure to at least one ACE (AOR = 1.60), two ACEs (AOR = 2.27), three ACEs (AOR = 2.88), and four or more ACEs (AOR = 2.80) were associated with higher likelihood of parent-reported SNAP benefits use in households during the past 12 months. This study underscores the need for comprehensive interventions to address children’s ACEs and strengthen public welfare policies for continuous use of SNAP benefits for families with limited resources in U.S households. Detailed implications for behavioral health, practice, and policy are further discussed.

## Introduction

Adverse childhood experiences (ACEs) pose a public health crisis linked to numerous negative outcomes, impacting individuals' overall physical, mental, behavioral, social well-being, and economic stability.^1–7^ Economic instability has consistently been linked to household food insecurity, with financially strained families facing a higher risk of limited access to food to mee their nutritional needs.^8,9^ In the United States (U.S), approximately 25% of people report having experienced at least three or more ACEs during their lifetime.^10^ ACEs refer to potentially traumatic events that occur before the age of 18. These events may include various forms of maltreatment, such as emotional, physical, and/or sexual abuse or neglect, peer violence, bullying, witnessing domestic or neighborhood violence, living with a family member struggling with substance abuse and mental health issues, experiencing parental separation or divorce, incarceration, household dysfunctions, and discrimination.^3,11–18^ Prior studies have indicated that having a history of ACEs can have significant long-term deleterious effects on children's physical and mental health, as well as nutrition-related behaviors that can increase the likelihood of food insecurity later in adulthood.^2,5,6,12,19–22^ The majority of children with ACEs come from households experiencing abject poverty, which increases dependence on social welfare programs for food security.^21,23,24^

In order to address food insecurity, the Supplemental Nutrition Assistance Program (SNAP) was established to provide important support to low-income families, reducing food insecurity and enhancing their diets.^25^ SNAP was officially established in 1964 and emerged as a national program in 1974. However, the origins of SNAP date back to the Great Depression, when surplus farm commodities were distributed; its focus has since evolved to support low-income families.^21,26^ Currently, SNAP is one of the most prominent social welfare programs used as a safety net to meet the nutritional needs of eligible low-income families in the United States (U.S). Eligibility for SNAP benefits depends on household income and resources, which must be below specific federal poverty guidelines. Applicants must also meet categorical requirements, such as U.S. citizenship or qualified non-citizen status, and comply with work-related conditions. States can add additional rules, but all must comply with federal regulations set by the U.S. Department of Agriculture.^21^ SNAP remains an important part of the U.S. social welfare systems, helping more than 40 million Americans and eligible non-citizens access food security and reduce poverty through monthly benefits that can only be used for food purchases.^28^ Nearly 90% of SNAP benefit recipients are eligible families with dependent children, the elderly, and individuals with disabilities.^28^ Currently, states distribute federal block grants to deliver SNAP benefits to low-income families; however, not all eligible U.S. households with children enroll in SNAP benefits.^2,21,25–31^

Studies have shown that exposure to ACEs increases the risk of household food insecurity, as children exposed to multiple adversities are significantly more likely to face limited or uncertain access to adequate food.^5,7^ Food insecurity is one of the main reasons many low-income families rely on SNAP benefits. This widespread public health problem is linked to poor nutrition that can increase the risk of failing health and psychosocial stress. Children and adults in food-insecure households are more likely to experience depression, anxiety, and other mental health issues compared to those in food-secure households.^32^ Food insecurity is strongly associated with  numerous educational problems and lower overall well-being, which intensifies existing health and socioeconomic disparities.^33^ Other studies have consistently associated specific types of ACEs with household food insecurity.^5–7,34^ For instance, economic challenges,^35,36^ parental incarceration,^37–43^ living with family member with mental health problems diagnosis,^44–46^ being orphaned,^47,48^ substance use problems in the households,^37,49^ exposure to violence,^6,7,19,50^ and discrimination,^51,52^ are evident risk factors connected to household food insecurity for low income families in the U.S. Simultaneously, the most recent study found that exposure to three or more ACEs led to an 8.14-fold increase in the relative risk of moderate-to-severe food insecurity for low income families.^6^

Several studies have also linked ACEs to food insecurity and other socioeconomic challenges in limited-resource settings.^5,6,19,20,29,53^ Research evidence suggests that SNAP is an important program that reduces health disparities by enhancing food security and improving health outcomes for low-income families with dependent children.^21,28,34^ Families with children with ACEs may be more likely to use safety net programs, such as SNAP benefits. This understanding is crucial for determining the appropriate level of support and designing interventions to mitigate the negative impact of ACEs and enhance food security in low-income families with dependent children, which is fundamental to the overall well-being and health of childhood development. Therefore, this study aims to expand this knowledge by examining ACEs and parent-reported use of SNAP benefits using a nationally representative sample of children in U.S. households, and specifically seeks to address the following research questions;

- What is the prevalence of children’s ACEs and parent-reported use of SNAP benefits in U.S. households?
- What specific types of ACEs experienced by children influence parent-reported use of SNAP benefits in U.S households?
- Is there a relationship between children’s cumulative ACEs and parent-reported use of SNAP benefits in U.S. households?

Given the multitude of evidence linking exposure to ACEs and food insecurity in low-income families,^2,5–7,19–21,25,28,29,53^, we hypothesized that children with ACE exposures would be more likely to come from households with parent-reported use of SNAP benefits after controlling for other characteristics*.*

## Methods

### Data Source

**Figure 1:**
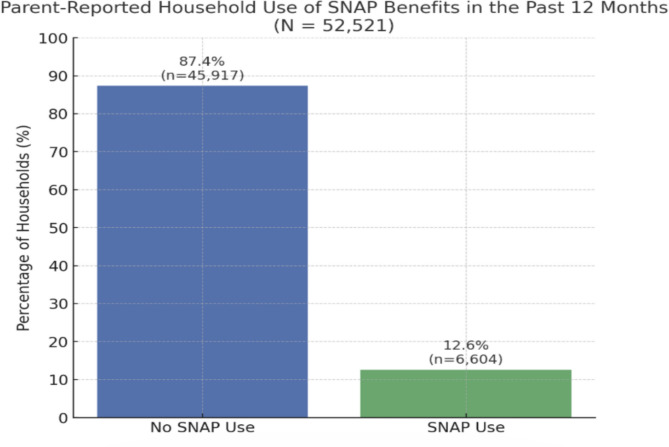
Parent-reported SNAP benefits use in U.S households (N = 52,521)

This study was a cross-sectional survey of non-institutionalized children aged 0 to 17 years, using data from the 2022 National Survey of Children’s Health (NSCH) in the U.S.^17^ The NSCH provides annual, state-level, nationally representative estimates about child health and healthcare indicators. Conducted by the U.S. Census Bureau for the U.S. Department of Health and Human Services, the Health Resources and Services Administration, and the Maternal and Child Health Bureau, the 2022 NSCH includes demographic characteristics, health status, healthcare access and utilization, functional status, neighborhood and community characteristics, early childhood issues, middle childhood and adolescence, as well as family functioning.^17^ Data for the 2022 NSCH were collected from July 8th, 2022, to January 20th, 2023. Parents or caregivers familiar with the child’s health participated in the study and responded on behalf of their children. The two-phase approach included (1) a household screener that identified children, demographics, and healthcare needs and (2) a detailed questionnaire completed by a parent or caregiver. Sampling weights were adjusted for nonresponse, and bias analyses confirmed decreased potential biases. In households with multiple children, one child was randomly selected.^17^ The weighted response rate was 37.4%, with an interview completion rate of 70.9%. Post-stratification adjustments accurately represented sociodemographic subgroups, resulting in an unweighted final sample of 54,103 children in U.S. households.^17^ In the current study, the authors only included samples of children whose parents responded to  using SNAP benefits in their households during the past 12 months (No/Yes). Therefore, the final sample consisted of 52,521 childrenwith parents reporting no use of SNAP benefits (n = 45,917; 87.0%) and use of SNAP benefits (n = 6,604; 12.6%) in U.S households (see Fig. 1).

### Measures

#### Dependent Variable

Parent-reported use of SNAP benefits was assessed by asking parents the following question: “During the past 12 months, even for just one month, did anyone in your family receive: food stamps or Supplemental Nutrition Assistance Program (SNAP) benefits?" Responses were dichotomously coded as either "No" (No SNAP use) or “Yes” (SNAP use)*.*

#### Independent Variables

*Adverse childhood experiences (ACEs)* were assessed by asking parents, to the best of their knowledge, whether their child had ever experienced any of the following: (1) difficulties covering basic needs like food or housing (financial hardship); (2) divorce/separation of parents or guardians; (3) death of a parent or guardian; (4) parent or guardian spending time in jail or prison; (5) living with someone who has alcohol/drug use problems; (6) living with someone who is mentally ill; (7) witnessed domestic violence; (8) being a victim or witness of neighborhood violence; (9) unfair treatment due to race; (10) unfair treatment due to health conditions; and (11) unfair treatment due to sexual orientation. This was dichotomously coded as “no” or “yes”. A composite measure of ACEs was created by summing all eleven items to determine the total number of ACEs experienced, which ranged from 0 to 11. Instead of using a continuous measure of ACEs, children were categorized into five groups: no ACEs, one ACE, two ACEs, three ACEs, or four or more ACEs. The study has adopted categories based on previous studies that used a similar approach to categorize ACEs.^34,53–55^

### Covariates

The assessed covariates were categorized according to the social-ecological framework model in the current study.^56^ Covariates were selected across key nested domains at the child, family, and community levels. Child characteristics included sex, age in years, and race/ethnicity. Family characteristics included the number of children in the households, household poverty/income level based on the Federal Poverty Level (FPL) threshold, parental education attainment, parental employment status, and family structure. Community characteristics included residence type and neighborhood conditions. Specific categories for each covariate used in the current study are shown in Table 1.

**Table 1:** Sample characteristics of children in U.S households whose parents reported SNAP benefit use (N = 6,604)

Variables	N	%
**Child characteristics**		
Child’s sex		
Male	3423	51.8
Female	3181	48.2
Child’s age (m = 8.6 years)		
0–5	2223	33.7
6–11	1228	18.6
12–17	1235	18.7
Child’s race/ethnicity		
White	4141	62.7
Black/African American	1228	18.6
Other	1235	18.7
**Family characteristics**		
Number of children in household		
1	2401	36.4
2	2176	32.9
3	1231	18.6
4 or more	796	12.1
Household poverty level		
Below 100%	3174	48.1
100—199%	2090	31.6
200—399%	1009	15.3
400% or above	331	5.0
Household education level		
Less than high school	581	8.8
High school	2239	33.9
Some college or associate degree	2341	35.4
College degree	1443	21.9
Parental employment (last 12 months)		
At least one parent employed	2773	67.4
No parents employed	1342	32.6
Family structure		
Two parents, married	1933	29.9
Two parents, not married	899	13.9
Single mother	2654	41.1
Other family type	973	15.1
Primary household language		
English	5703	87.0
Spanish	586	8.9
Other	267	4.1
**Community characteristics**		
Place of residence		
Non-metropolitan	2204	37.9
Metropolitan	3611	62.1
Neighborhood poorly kept/rundown housing		
No	5190	79.7
Yes	1322	20.3

*Sample characteristics were based on parents’ reports on SNAP benefit  use during the past 12 months. N = unweighted frequencies. %* = *Weighted percentages*

### Data Analysis

Before conducting data analysis, weights were applied to ensure that the sample accurately represents the target population by adjusting for unequal selection probabilities, nonresponse, or demographic differences in the data. This allows estimates such as means, percentages, and totals to be generalized to the larger population. Descriptive statistics were then performed to generate unweighted frequencies and weighted percentage distributions across nominal variables. Equally important, means and standard deviations were calculated for continuous or numerical variables. For descriptive statistics, only children whose parents reported having used SNAP benefits in their households during the past 12 months were included in the analysis (N = 6,606).

On the other hand, binary logistic regression was used to determine significant associations because the outcome was a binary variable. Logistic regression analyses were conducted in two stages to examine the association between children’s ACEs and parent-reported use of SNAP benefits in households during the past 12 months. In Stage 1, two models of logistic regression were used to assess the significant association between each individual ACE type and parent-reported use of SNAP benefits during the past 12 months. In Model 1, each ACE type was included in the model independently as a predictor of SNAP use during the past 12 months without adjusting for covariates. The unadjusted model provided a baseline measure of the direct association between ACEs and SNAP use during the past 12 months, without accounting for potential confounders. In Model 2, all the covariates at the child, family, and community levels were included. The adjusted model shows a strong predicted direct association between children’s ACEs and SNAP use during the past 12 months, even after controlling for other confounding influences. In stage 2, cumulative ACE scores were analyzed as predictors of SNAP use during the past 12 months involving four hierarchical logistic regression models. Model 1 determined the unadjusted odds ratios (ORs) and 95% confidence intervals (CIs) without the inclusion of covariates. Model 2 controlled for child characteristics, while Model 3 included both child and family characteristics. The final Model 4 retained all the covariates in models 1 and 2 (child and family characteristics) and included community characteristics as covariates. The step-by-step addition of covariates in the adjusted models helped to assess potential confounding, resulting in more accurate estimates of the association between children’s ACEs and use of SNAP benefits in households during the past 12 months. This approach separated the direct impact of ACE exposure from demographic, socioeconomic, and contextual influences. In the current study, all the results are presented as both unadjusted (crude) odds ratios (ORs) and adjusted odds ratios (aORs) with 95% confidence intervals (C.I.). The significance level was set at a p-value of 0.05. All data analyses were performed using SPSS version 29.0.^57^

### Ethical Consideration

This study used 2022 NSCH data, which is publicly available. As a result, this study was exempted from ethical review by the University of Cincinnati. Additionally, the requirement for informed consent was also waived, as this was not applicable due to the use of secondary data in this study.

## Results

### Sample Characteristics

Table 1 shows the sample characteristics of children as reported by parents. Among the 52,521 children in households included in the sample, only 12.6% (n = 6,604) of parents reported using SNAP benefits in their household during the past 12 months (see Fig. 1). Out of 6,604 children whose parents reported using SNAP benefits in their households during the past 12 months, more than half were boys (51.8%), with an average age of 8.6 years, ranging from 6 to 17 years. Nearly two-thirds of the children were white (61%). However, fewer than half of these children lived in households with at least one other child (44.4%) and lived in households where the poverty threshold ratio was below 100% (36.8%). In terms of family characteristics, children came from households where parents reported having completed at least a bachelor’s degree or higher (38.8%) and were headed by single mothers (43.8%). However, a good majority of children lived in households where one parent was employed (69.7%), in poorly maintained or rundown housing conditions (71.6%), and primarily lived in metropolitan or urban areas (60.2%).

### Prevalence of Children’s ACEs and Parent-Reported Use of SNAP Benefits in U.S Households

Table 2 shows the prevalences of children’s ACEs and parent-reported use of SNAP benefits in households during the past 12 months. Out of the 6,604 children with parents who reported using SNAP benefits in their households during the past 12 months, the prevalence of parent-reported SNAP benefit use was higher for children with specific ACE types in U.S households. Notably, 41% of children who had parents/guardians who were divorced, 34.8%  of children  who experienced economic hardship, 17.6% of children who lived with a family member with substance use problems, and 17% of children who lived with someone with mental illness, lived in households where parents reported using SNAP benefits during the past 12 months. Similarly, 13% of children who had at least one parent/guardian who spent time in jail,  prison or witnessed neighborhood violence, 9.2% of children who were victims of violence, 5.8% of children who experienced racial discrimination, 5.6% of children who experienced discrimination based on health conditions, and 3.1% of children who experienced discrimination based on sexual orientation, lived in households where parents reported using SNAP benefits during the past 12 months.

**Table 2:** The prevalence of children’s ACEs and parent-reported SNAP benefits use in U.S households (N = 6,604)

Variables	N	%
**Individual ACEs**		
Family economic hardship		
No	4488	65.2
Yes	2285	34.8
Parent/guardian divorced		
No	3787	59.0
Yes	2635	41.0
Parent or guardian died		
No	6024	93.9
Yes	391	6.1
Parent or guardian spent time in jail/prison		
No	5289	82.5
Yes	831	13.0
Victim of violence		
No	5808	90.8
Yes	585	9.2
Witnessed neighborhood violence		
No	5558	87.0
Yes	831	13.0
Lived with a family member with mental illness		
No	5302	83.0
Yes	1083	17.0
Lived with a family member with substance use problem		
No	5261	82.4
Yes	1125	17.6
Discriminated due to their race		
No	6031	94.2
Yes	369	5.8
Discriminated due to health conditions		
No	6065	94.4
Yes	362	5.6
Discriminated due to sexual orientation		
No	4095	96.9
Yes	133	3.1
**Cumulative ACEs**		
0 ACEs	2746	42.5
1 ACE	1614	25.0
2 ACEs	811	12.5
3 ACEs	557	8.6
4 ≥ ACEs	740	11.4

*Cross tabulations were performed to obtain the prevalence estimates between individual ACEs and Use of SNAP benefits during the past 12 months*

Conversely, children without ACEs (42.5%) were more likely to live in households where parents reported using SNAP benefits during the past 12 months than those with one ACE (25%), two ACEs (12.5%), three ACEs (8.6%), and  four or more ACEs (11.4%). Overall, 57.5% of children who experienced at least one or more ACEs lived in households where parents reported using SNAP benefits during the past 12 months.

### Association Between Children’s Individual ACE Types and Parent-Reported Use of SNAP Benefits in U.S Households

Table 3 presents the association between children’s individual ACEs and parent-reported use of SNAP benefits in households during the past 12 months. In the adjusted regression model involving individual ACE types, specifically children who experienced family economic hardships (aOR = 2.29; 95% CI = 1.69–3.10, p < 0.001) and were discriminated based on their health conditions (aOR = 1.69; 95% CI = 1.09–2.63, p < 0.05) were more likely to come from households where parents reported using SNAP benefits during the past 12 months. In contrast, children who had lived with family members with mental illness were less likely to come from households where parents reported using SNAP benefits during the past 12 months compared to those who did not (aOR 0.70; 95% CI = 0.50–0.99, p < 0.05). However, children with at least one parent or guardian who had died were more likely to come from households where parents reported using SNAP benefits during the past 12 months only before covariate adjustments (OR 0.78; 95% CI = 0.78–1.31, p < 0.05).

**Table 3:** Association between children’s individual ACEs and parent-reported SNAP benefits use in U.S households

Variables	Model 1	Model 2
**Individual ACEs**	**OR (95% CI)**	**AOR (95% CI)**
Family economic hardship	2.64 (2.04–3.43)***	2.29 (1.69–3.10)***
Parent or guardian divorced	1.30 (1.00–1.71)	1.40 (.100 −1.97)
Parent or guardian died	.58 (.38 –.89)*	.64 (.40—1.04)
Parent or guardian spent time in jail/prison	1.01 (.78–1.31)	1.11 (.79–1.58)
Victim of violence	1.09 (.84–1.41)	1.04 (.70–1.55)
Witnessed neighborhood violence	.99 (.75–1.310)	1.03 (.70–1.52)
Lived with mentally ill person	.82 (.63–1.07)	.70 (.50—.99)*
Lived with person with alcohol/drug problems	.78 (.60–1.01)	.80 (.55–1.16)
Discriminated due to race	.71 (.50–1.02)	.76 (.50–1.15)
Discriminated due to health conditions	1.37 (.94–2.00)	1.69 (1.09–2.63)*
Discriminated due to sexual orientation	.76 (.42–1.37)	.67 (.35 −1.30)

^*^ = *P* < .05, ** = *P* < .01, **^*^ = *P* < *.001, CI* = *Confidence interval, OR* = *Odds ratio, AOR* = *Adjusted odds ratios. Reference categories include participants who never experienced any of the individual ACEs. Model 1* = *unadjusted bivariate logistic model was performed between ACEs and SNAP benefits use, adjusted for child, family, and community characteristics*

### Association Between Cumulative Children’s ACEs and Parent-Reported Use of SNAP Benefits in U.S Households

Table 4 presents the results of the association between children’s cumulative ACEs and parent-reported use of SNAP benefits in households during the past 12 months. In Model 1, children who experienced at least one ACE (aOR = 2.53; 95% CI = 2.37–2.71, p < 0.001), two ACEs (aOR = 3.74; 95% CI = 3.43–4.09, p < 0.001), three ACEs (aOR = 5.33; 95% CI = 5.81–7.06, p < 0.001), and four or more ACEs (aOR = 6.41; 95% CI = 5.81–7.06, p < 0.001) were more likely to come from households where parents reported use of SNAP benefits during the past 12 months compared to those without ACEs. These significant effects were partially attenuated by including child, family, and community-level characteristics in Models 2, 3, and 4 (covariates). Despite this, these findings remained significantly consistent across Models 1, 2, and 3.

**Table 4:** Association between children’s cumulative ACEs and parent-reported SNAP benefits use in U.S households

Variables	Model 1	Model 2	Model 3	Model 4
**Cumulative ACEs**	OR (95% CI)	AOR (95% CI)	AOR (95% CI)	AOR (95% CI)
No ACEs	Ref	Ref	Ref	Ref
1 ACE	2.53 (2.37–2.71)***	2.60 (2.42–2.78)***	1.59 (1.43—1.77)***	1.60 (1.43—1.79)***
2 ACEs	3.74 (3.43–4.09)***	4.04 (3.68–4.43)***	2.24 (1.94—2.58)***	2.27 (1.95—2.65)***
3 ACEs	5.33 (4.79–5.93)***	5.99 (5.36–6.71)***	2.94 (2.45—3.53)***	2.88 (2.36–3.51)***
4 or more ACEs	6.41 (5.81–7.06)***	7.42 (6.69–8.22)***	2.81 (2.37—3.34)***	2.80 (2.32—3.38)***

^*^ = *P* < .05, ** = *P* < .01, **^*^ = *P* < *.001, CI* = *Confidence interval, OR* = *Odds ratio, AOR* = *adjusted odd ratios. Ref* = *Reference category. For Individual ACEs, Model 1 is an unadjusted bivariate logistic regression performed between ACEs and SNAP benefits use, while Model 2 adjusts for child, family, and community characteristics simultaneously. For cumulative ACEs, Model 1* = *unadjusted bivariate logistic was performed between ACEs and SNAP benefits use, Model 2* = *adjusted for the child’s characteristics, Model 3* = *adjusted for the child and family level characteristics, and Model 4* = *adjusted for the child, family, and community characteristics*

In the fully adjusted model (Model 4), particularly children who experienced at least one ACE (aOR = 1.60; 95% CI = 1.43—1.79, p < 0.001), two ACEs (aOR = 2.27; 95% CI = 1.95 −2.65, p < 0.001), three ACEs (aOR = 2.88; 95% CI = 2.36—3.51, p < 0.001), and four or more ACEs (aOR = 2.80; 95% CI = 2.32 −3.38, p < 0.001) were more likely to come from households where parents reported using SNAP benefits during the past 12 months compared to those without ACEs.

## Discussion

The current study assessed the prevalence of ACEs and their association with parent-reported use of SNAP benefits in households using a nationally representative sample of children in the United States (US). Approximately 57.5% of children with at least one or more ACEs came from households where parents reported having used SNAP benefits. Specifically, children who had experienced their parents’ separation or divorce, had family members with mental health issues, and encountered economic hardships lived in households where parents reported higher rates of SNAP benefit use. With the increasing divorce rates in the U.S,^58^ there has been a sharp increase in single-parent households, resulting in financial pressures that limit their access to resources necessary for survival compared to children in two-parent households.^59,60^ At the same time, families dealing with economic hardships and mental health problems often encounter higher food insecurity levels because of limited finances and reduced ability to work, making them more dependent on food assistance programs, such as SNAP benefits.^9,25^

In line with numerous previous studies that have linked various dimensions of poverty to food insecurity,^5,6,9,19,20,25,29,61–63^ the current study found that children experiencing economic hardship were more likely to come from households that used SNAP benefits. Financial constraints enable families with dependent children to rely on essential safety nets, such as SNAP benefits, to ensure that there is enough food supply while managing other financial burdens, unstable housing, unemployment, medical debt, and other perceived challenges.^32,34,53,64–66^ These economic threats can pose an enormous potential challenge to children with ACEs, especially in homes where food insecurity is evident. The effects of economic challenges within families can further undermine their ability to maintain stable employment and income, thereby increasing the risk of food insecurity in households.^55,56^ This may compel families with limited resources to continuously seek SNAP benefits to meet their household's nutritional needs essential for survival. As families become increasingly food insecure, safety net programs like SNAP benefits provide relief.^9,25,31,32,67^ These safety net programs support families with children who have experienced ACEs and other challenging behaviors, making them more reliant on food assistance programs such as SNAP benefits in the U.S.

Equally important, children who were discriminated against based on their health conditions were more likely to come from households with higher food insecurity, as evinced by parent-reported use of SNAP benefits. Health complications often lead to increased medical expenses, such as hospital visits, medications, and treatments, thereby diminishing household resources for food and other necessities.^68,69^ The burden of managing physical health conditions can result in significant psychological distress, including anxiety and depression, which may diminish individuals' motivation and capacity to seek additional resources.^70^ Poor health conditions may further strain the finances of affected families, leading to increased food insecurity, as they could be forced to choose between covering essential healthcare costs and meeting their nutritional needs. In these challenging circumstances, families with dependent children may enroll to receive SNAP benefits, which have been shown to combat food shortages effectively for eligible family members.^2,21,28–31^ Thus, there is a need to increase access to SNAP benefits for families with dependent children with poor health conditions, which provides numerous nutritional advantages for maintaining optimal health during childhood development and enhancing the overall quality of life.

Previous studies have identified a stronger association between mental health issues and food insecurity.^9,44–46,67^ This contradicts the study's’ findings that children who lived with a family member experiencing mental health issues were less likely to come from households that used SNAP benefits. This may suggest that mental health is not necessarily a direct path to food insecurity. Although the current study found no direct link, this can be argued that mental challenges may create insurmountable barriers in navigating complex bureaucratic social welfare systems. Mental illness often carries a social stigma, which can lead to social isolation,^71,72^ diminish affected individuals’ ability to access social support networks that might otherwise assist them in applying for benefits like SNAP, and limit their ability to stay informed about government social welfare programs.^25^ Even if families are eligible for receiving SNAP benefits, they may be reluctant to apply due to concerns about being judged or perceived as dependent on public aid, especially if they have already experienced social stigma related to mental health issues. Therefore, families dealing with mental health challenges may be less likely to seek social safety net programs such as SNAP benefits, even if they are confronting chronic food insecurity in their households.

Most importantly, as evidenced in the current study, children with a higher number of ACEs were more likely to come from households where parents reported use of SNAP benefits. This supports the current study'shypothesis and aligns with other previous studies connecting ACE exposures to food insecurity and the need for overarching support from government social welfare programs.^2,5,6,19–21,34,53,63^ ACEs may intricately co-occur with relative conditions of poverty, marked by economic instability, which can expose families to ongoing food insecurity due to limited resources. The cyclical nature of poverty indicates that children experiencing ACEs might grow up in environments where dependence on SNAP benefits may be a common norm, thus maintaining reliance on government assistance programs as an essential resource for meeting their nutritional needs. As these children transition into adulthood, they may be more likely to continue relying on SNAP benefits to combat food insecurity in their future households, just as their parents did. Thus, it is crucial to prevent any form of adversity that could be possible negative indicators of increased food insecurity and prevent the perpetuation or cyclical nature of ACEs and dependence on SNAP benefits for families with limited resources in U.S households. Although SNAP benefits continue to lift millions of Americans out of food insecurity,^21,25–28^ more robust efforts should be directed toward identifying long-term interventions to address both ACEs and other environmental indicators of perceived food insecurity for families with dependent children with limited resources who may be constantly dependent on SNAP benefits in the U.S.

## Implications for Behavioral Health

This study provides numerous important implications for behavioral health, particularly regarding children’s exposure to ACEs, which warrants attention. These findings underscore the need for behavioral health systems to adopt a trauma-informed approach that acknowledges the impact of early adversity on children’s overall well-being and development. This includes integrating routine ACEs screening into school-based health and pediatric services to identify at-risk children early and provide them with the necessary mental health and social support.^73^ Behavioral health providers must receive training to address the specific needs of families with children experiencing food insecurity and family instability, employing culturally sensitive and developmentally appropriate interventions in limited resource settings.^74^ Behavioral health trainings not only improve the quality of care at the local level but also strengthen the behavioral health workforce nationally by ensuring consistent, evidence-based practices that promote equitable mental health outcomes across diverse populations in the U.S.

Furthermore, social stigma and discrimination, particularly concerning mental health and chronic illness, must be confronted through public education and policy to guarantee equitable access to care.^75^ Behavioral health professionals, educators, and social service providers must collaborate to establish comprehensive support systems that address both the psychological and material needs of vulnerable families with dependent children in resource-constrained settings. These collaborative behavioral health prevention interventions can enhance long-term outcomes for children and reduce reliance on public assistance programs, such as SNAP benefit use, ultimately promoting healthier and more resilient communities in the U.S.

### Implications for Practice

This study has shown that children’s exposure to ACEs is significantly associated with household use of SNAP benefits in limited-resource settings in the U.S. In order to address both the root causes of ACEs and food insecurity, holistic interventions to reduce the long-term social and economic consequences for families with limited resources are needed. For instance, state and local governments, alongside service providers, should prioritize the development of programs that combine SNAP benefits with mental health services, financial literacy workshops, and other holistic support initiatives. These social welfare oriented programs can help address underlying issues such as economic challenges and family instability, which contribute to food insecurity for families with dependent children. Social welfare agencies that work directly with families in need should also prioritize ACE prevention education for families in resource-limited households in the U.S. Implementing trauma-informed approaches in all social welfare programs, including SNAP benefits, can ensure that the needs of families affected by ACEs are appropriately met. This includes training caseworkers and other program staff on understanding the negative effects of ACEs and how to provide compassionate and non-judgmental support to children and their families. By implementing a trauma-informed approach in delivering welfare programs, families may feel more at ease seeking assistance, ensuring that the services provided are attuned to those experiencing the aftermath of childhood trauma. Early prevention of ACEs can alleviate the negative impacts of ACEs on children's development, mental health, and overall well-being,^76^ ultimately decreasing the necessity for long-term support like SNAP benefits. This may also involve routine ACE screenings in healthcare and school environments, which can help identify at-risk children and prompt timely interventions, such as mental health support, parent training, and family counseling services.

Furthermore, community agencies and other service providers can also strengthen community support networks, such as peer groups and outreach programs, to help families with children who have experienced ACEs and might be at risk of food insecurity in navigating public welfare services like SNAP benefits. This should include helping families with children to understand their rights, apply for SNAP benefits, and access other social services. Addressing the root causes of ACEs, such as economic instability and family dysfunction, could potentially reduce future reliance on social welfare services, breaking the cycle of food insecurity and poverty. Despite this, nutritional assistance programs such as SNAP should be strengthened to provide ongoing support for families with dependent children who have limited resources.^25,27^ This may involve a collaborative effort among service providers, including health, social services, and education sectors, which is essential for addressing the complex needs of families with dependent children and limited resources. Addressing discriminatory barriers to accessing public assistance is crucial, especially for children who have experienced discrimination due to health conditions, as demonstrated in this study. Social welfare staff should receive training to recognize and combat stigma and discrimination, and ensure that families with dependent children obtain the support they need to meet essential survival needs such as food. While promoting self-sufficiency is important, ensuring access to SNAP benefits is equally important, as they provide essential support for families at risk of food insecurity in resource-limited in the U.S households.^21,25–28^

### Implications for Policy

In order to mitigate ACEs and their negative  impact on food insecurity, which increases SNAP use for U.S households as evidenced in this study, policymakers should focus on some crucial areas. Policymakers can further enhance access to SNAP benefits by implementing automated eligibility assessments and providing application assistance, which could help remove bureaucratic obstacles for vulnerable families with dependent children who require food assistance in their households. Promoting healthcare services, expanding healthcare coverage, and enforcing anti-discrimination policies can be effective strategies to address the interconnected issues of food insecurity and health disparities, particularly affecting children from families experiencing discrimination due to poor physical health, which can be addressed through integrating violence prevention strategies into broader food assistance programs. This integration can enhance economic stability, increase food security, and help prevent ACEs for children in households with limited resources in the U.S.

Social welfare agencies should promote social policies to reduce violence in high-poverty communities, as this contributes to food insecurity and the need for SNAP benefits. Advocating for policy changes to simplify SNAP access for families with a history of ACEs is essential, including streamlining applications, reducing stigma, and providing accessible application options, especially for those experiencing mental health or substance abuse challenges. A potential decrease in barriers to accessing SNAP enrollment  can ensure that eligible families with low income, particularly those with children with ACEs, can access food in their households with limited resources in the U.S.

### Limitations of the Study

The current study has several limitations that warrant attention and consideration. The cross-sectional design of the NSCH limits the ability to draw causal inferences between children’s ACEs and parent-reported use of SNAP benefits in households during the past 12 months, as data were collected at a single point in time. SNAP benefits use was assessed based on parental reports during the past 12 months, which may not accurately capture long-term or consistent use patterns for households in U.S. For instance, families may have experienced changes in eligibility or participation over time, which could introduce potential misclassification bias and inaccuracies in the information that was reported in the current study.

In the current study, the measurement of ACEs did not include other important adversities such as maltreatment which are part of the traditional ACE framework including sexual abuse, emotional abuse, physical abuse and neglect (i.e., physical and emotional abuse). As a result, the total ACE exposure captured in this dataset may underestimate the full scope of children’s adverse experiences, which many studies use, and may reduce comparability with other studies due to different measurement methods being applied. Additionally, because the data were parent-reported, social desirability biases and the ability to recall all the significant events could affect accuracy of information being reported, particularly for sensitive topics such as family adversity or use of SNAP benefits during the past 12 months. The use of secondary data also limited the inclusion of certain contextual variables, such as parental mental health, community support systems, and family coping strategies, that might help explain the observed relationships. Moreover, a dichotomous measure for parent-reported use of SNAP benefits during the past 12 months may oversimplify the complexity of exposure intensity and program involvement for families with dependent children in U.S households.

Despite the identified limitations, this study also highlights several strengths that were employed to mitigate potential bias and enhance the study's rigor and generalizability. Using a large, nationally representative dataset (NSCH) increased the applicability of the findings to diverse U.S. households. Survey weights were applied to correct for sampling design and non-response bias, ensuring that the results accurately reflect the population. Additionally, including multiple covariates across child, family, and community characteristics helped control for confounding factors and boosted the reliability of the results for the general population. Future studies using longitudinal and mixed methods are recommended to explore more detailed and time-sensitive connections between ACEs and families’ use of SNAP benefits in the U.S households with limited resources.

## Conclusion

This study shows that children’s ACEs are associated with parent-reporteduse of SNAP benefits during the past 12 months. Particularly, children who experienced specific ACE types such as economic hardships and health discrimination were more likely to come from households were parents reported use of SNAP benefits during the past 12 months. In contrast, children who lived in households with  a family member with mental health issues were less likely to come from households where parents reporteduse of SNAP benefits during the past 12 months. Therefore, this study underscores the need for a comprehensive range of interventions to identify the root causes of ACEs particularly forchildren  inlimitedresource families, promote education on ACE prevention and trauma-informed strategies, and strengthen public welfare policies to ensure adequate SNAP support for families with dependent children in U.S households with limited resources.

## Data Availability

The data used for the study will be available upon request from the corresponding author.

## Declarations

### Dual Publication

Not applicable. This study has not been published elsewhere.
